# Hernia Cerebri

**Published:** 1889-11-16

**Authors:** 


### SURGERY.

### HERNIA CEREBRI.

Hernia cerebri, or protrusion of brain matter, usually mixed with inflammatory products, is very often the result of a wound through the dura mater. A standard author says it is probably always associated with some suppuration of the brain. In a recent number of the *Philadelphia Therapeutic Gazette*, Dr. G. Archie Stockwell, of Port Huron, has some remarks on the subject of the treatment of hernia cerebri. Detailing a case of injury to the brain, he continues, "Suddenly hernia cerebri appeared, and the medical attendant, following the teachings of his *alma mater's* text-books, feared to resort to any measures of relief except the authorised repression by pressure." The autopsy revealed two fluid ounces of pus less than an inch from the opening of the wound, and consequently easily and safely accessible to the knife. "But because the pus was surrounded by cerebral tissue the patient must needs be allowed to die in obedience to an absurd fetich." According to the author, hernia cerebri is invariably one of three conditions. First, a tumour composed wholly or partially of disorganised brain substance; second, brain matter protruded by inflammatory processes; third, exuberant or excessive granulations. Now, if the hernia is composed of disorganised tissue, the economy derives no benefit therefrom, and the sooner it is disposed of the better. If the protrusion is the result of inflammatory processes, it is *prima facie* evidence of danger lurking behind, probably due to accumulated purulent fluid, and common-sense dictates that an outlet should be afforded. Exuberant granulations are repressed by the free employment of the scalpel or caustics in accordance with indications that demand their use in other parts of the body, and it is a logical sequence that such should not be ignored or neglected merely because the tissue is cerebral. But by teachers and textbooks generally hernia cerebri is regarded as a *noli me tangere.* And the author especially comments on the dictum of Prof. C. Nancrede, of Philadelphia, who says "interference must be avoided, especially when granulations are springing upon or around the protuberance, as we thus remove pressure and encourage growth." But "atop of all this," says Dr. Stockwell, "he insists on resort to compression, with the view to forcing the mass back upon its matrix." Now the treatment of hernia cerebri has long been a subject of some disputation. Some surgeons are in the habit of applying pressure, others cut off the projecting mass, or ligature it. Successful cases are on record by the army surgeons of the Peninsula War after all these methods of treatment. But most surgeons of the present day consider the best practice is in doing very little. We quite agree, however, with the author that if there is pus in the brain it should be evacuated. But how is this to be discovered? We do not altogether agree with Dr. Stockwell where he says that hernia cerebri depends "upon pressure consequent on accumulations of inflammatory and consequently of effete products." There are quite as many cases of recovery on record without operative interference as the reverse, and, notwithstanding all Dr. Stockwell advances, we certainly advocate expectant treatment.

We are altogether inclined to agree with Mr. Prescott Hewett and Mr. J. W. Hulke, who, in their joint article on injuries of the head, in the first volume of "Holmes' System of Surgery," say the treatment of hernia cerebri is to be of the simplest kind. The less the protrusion is meddled with the better. As a general rule, removal either by tearing away, slicing off, or ligature is to be avoided. "The parts are to be kept as clean as possible, for which purpose gentle syringing with some slightly astringent antiseptic lotion may be resorted to, and an antiseptic absorbent dressing should be put on." We do not, however, agree with what the authors named, further proceed to observe. They remark, "in the earlier stages, when the tumour is but small, general pressure may be advantageous." We do not believe that general pressure applied to the brain will ever be advantageous in any condition. The authors referred to admit that "in larger protrusions it should be abandoned." The brain is not an organ which may be compressed with impunity, even when in a healthy condition, and when bordering on a state of inflammation, as is the case when hernia cerebri appears, it is much less likely to tolerate pressure.

